# ‘The world is not only for hearing people – It’s for all people’: The experiences of women who are deaf or hard of hearing in accessing healthcare services in Johannesburg, South Africa

**DOI:** 10.4102/ajod.v10i0.800

**Published:** 2021-07-20

**Authors:** Khetsiwe P. Masuku, Nomfundo Moroe, Danielle van der Merwe

**Affiliations:** 1 Department of Speech Pathology, Faculty of Humanities, University of the Witwatersrand, Johannesburg, South Africa; 2 Department of Audiology, Faculty of Humanities, University of the Witwatersrand, Johannesburg, South Africa; 3 Department of Speech Pathology and Audiology, Faculty of Humanities, University of the Witwatersrand, Johannesburg, South Africa

**Keywords:** access, healthcare, women, deaf, South Africa

## Abstract

**Background:**

Despite legal and adopted frameworks purporting access to healthcare and rehabilitation services, which are both a human right and key to developmental issues, women who are deaf and/or hard of hearing (HoH) are still excluded and experience barriers when accessing healthcare services. Largely, this is attributed to communication barriers between healthcare professionals and women who are deaf and/or HoH. There have been limited research studies carried out on women with invisible disabilities, such as deafness, especially amongst African women.

**Objectives:**

This study sought to gain insights into the communication experiences of women who are deaf or HoH when accessing public healthcare services in hospitals in Johannesburg.

**Methods:**

A qualitative research study employing semi-structured interviews with 10 African women who are deaf and/or HoH residing in Johannesburg, South Africa and attending government healthcare facilities was conducted. Participants were purposively selected. Data were analysed using thematic analysis.

**Results:**

Data revealed the following themes: communication barriers resulting in compromised quality of care and infringement on participants’ right to confidentiality; accommodation that is not accommodative and negative attitudes of healthcare professionals.

**Conclusion:**

The findings of this study confirm the alienating, exclusion, marginalisation, discrimination, invisibility, lack of independence and autonomy of women who are deaf and/or HoH when accessing healthcare services. Therefore, this study argues for a need for the conscientisation of healthcare professionals on communication needs of persons who are deaf and/or HoH. This has implications for the implementation of training programmes that will address communication, reasonable accommodation and attitudes of healthcare professionals.

## Introduction

Enjoyment of the highest attainable standard of health is one of the fundamental rights of every human being (Kuenburg, Fellinger & Fellinger 2016:1). However, this is not always the case for black deaf women globally. The World Health Organization (WHO) maintains that access to healthcare and rehabilitation services are both a human right and key to development issues (World Disability Report 2011). Relatedly, the South African constitution also states that all people living in South Africa have the equal right to accessing healthcare services and should not be discriminated against on the basis of their language, gender, race or disability (Gutto 2001). In 2007, the South African government ratified the United Nations Convention on the Rights of People with Disabilities (CRPD) (Flynn 2011), emphasising its commitment to ensuring that the rights to access to healthcare for persons with disabilities are upheld and secured. As a means of operationalising the CRPD, the South African government enacted the White Paper on the rights of persons with disabilities (Department of Social Development 2016). The mandate of the White Paper on the rights of persons with disabilities was the removal of discriminatory barriers that perpetuate the exclusion and segregation of persons with disabilities in all spheres of society, including access to healthcare services (Department of Social Development 2016).

### Access to health care

The South African Department of Public Service Administration (2009) defined access as all people having equal opportunities and availability of services or products from which they can benefit, regardless of their social class, ethnicity, background or physical disabilities. The concept of ‘access’ is strongly tied to the concept of human rights (South African Department of Public Service Administration 2009). South African Department of Public Service Administration (2009) further stated that accessibility also speaks to physical aspects of being able to access resources from public facilities or being able to work in public facilities without experiencing any barriers regardless of mobility and sensory impairments. Different authors have presented different dimensions of access, for example, Penchansky and Thomas (1981) posited that access is optimised when the following dimensions are accounted for: accessibility, availability, acceptability, affordability and accommodation. Peters et al. (2008), however, argued that in order for access to be achieved, availability, acceptability, geographical accessibility and financial accessibility need to be considered. According to the Department of Public Service (2009), access is defined according to the following aspects: availability which relates to access to the physical environment, affordability which is economic access, and acceptability which relates to socio-cultural access of services that meet a minimum standard equality. Whilst there are numerous definitions of access to healthcare, there is consensus that access to healthcare encompasses the availability of services at the particular time they are needed.

Healthcare is not easily accessible to the majority of South Africans, and legislation does not guarantee that human rights will be upheld (Meyer 2010). In order to substantiate this argument, Meyer (2010) highlighted the inequalities in the service delivery between public and private healthcare facilities. Pieterse (2014) also attested that the majority of the South African population is subjected to an overburdened, understaffed, underequipped and under-resourced public healthcare system.

Despite all the policies and legislation implemented to address these challenges, access to healthcare services for marginalised and vulnerable groups remains a challenge, particularly for persons with disabilities in developing countries (Eide et al. 2015; Vergunst 2016; World Disability Report 2011). Both internationally and locally, there is a plethora of literature documenting barriers to accessing healthcare experienced by persons with disabilities, specifically in the low- and middle-income countries (LMIC) (Ali et al. 2013; Eide et al. 2015; Harris et al. 2020; Varela et al. 2019; Vergunst 2016; Vergunst et al. 2015; Vergunst et al. 2017). These barriers include poor staffing (Masuku 2020), inadequate training of healthcare professionals (World Disability Report 2011), lack of budgets for provision and maintenance of assistive devices (Matter & Eide 2018), transportation challenges and costs (Gudlavalleti et al. 2014; Masuku 2020; Syed, Gerber & Sherp 2013), negative attitudes of healthcare professionals (Masuku 2020; Moroe 2014; Vergunst 2016; World Disability Report 2011), physical access (Vergunst et al. 2015), communication barriers (Kritzinger et al. 2014; Masuku 2019; Moroe & De Andrade 2018a, 2018b) and gender inequalities (Evans et al. 2001; Groener 2013).

The challenges of accessing healthcare services are exacerbated, particularly for women living in patriarchal societies (Howell, Chalklen & Alberts 2006; Shastri 2014; Smith, Braunack-Mayer & Wittert 2006; Verdonk, Mans & Lagro-Janssen 2006), and further compounded in women who are deaf. Ubido, Huntington and Warburton (2002) lamented that women who are deaf do not have an equal access to healthcare services because of a variety of barriers, which are not only on the basis of their gender and disability but also on the basis of language oppression. The source of these challenges is rooted on ‘acceptability’, which relates to socio-cultural access of services that meet a minimum standard equality, what Kritzinger (2011) terms the ‘attitude’ of healthcare professionals and the hearing community. Deaf women face communication barriers when interacting with hearing healthcare professionals, which, in turn, results in inadequate service delivery and poor access to much needed services.

### Deafness in South Africa

Historically, people who are deaf have been marginalised and discriminated against both internationally and locally (Tye-Murray 2009). One of the reasons for this marginalisation potentially stems from the fact that deafness in itself is an invisible disability (Tye-Murray 2009). Hence, it is often silenced, unrecognised, ignored and even forgotten by the hearing community (McDougall 2006; Purcell 2014). Power and Leigh (2003) and Napier (2002) argued that deafness is primarily a ‘communication disability’, meaning if deaf people were provided access to information and means to communicate with the hearing community, they would not be regarded as having a disability. Despite the many studies lobbying for the formalisation of South African Sign Language (SASL) and interpreting services for deaf people in South Africa (Aarons & Akach 2002; Beukes 2009; Glaser & Van Pletzen 2012; Moroe 2014; Reagan 2008; Selzer 2010), sign language is not yet afforded the same status as other languages spoken locally (Morgan, Glazer & Magongwa 2016). According to Magongwa, approximately 500 000 deaf people use sign language in South Africa. Haricharan et al. (2013) argued that the rights of South Africans who are deaf are violated when their communication needs, including the need for a professional SASL interpreter, are neglected. Because of the unavailability of healthcare professionals who are able to communicate in SASL and qualified SASL interpreters, deaf people cannot exercise their independence in accessing healthcare services, and consequently, they are left to depend on their family members or people waiting in the queue to help them (Heap & Morgan 2006).

Aarons and Akach (2002) acknowledged that in South Africa, deaf women are a minority and are marginalised, with black women being the most marginalised of all. Currently, there are a dearth of studies that have been carried out on the experiences of South African deaf women to access healthcare services. Reportedly, persons with disabilities in South Africa generally lack access to or knowledge of basic health and social services (Barratt 2007). Globally, research studies have focused primarily on ‘first world’ contexts rather than on deaf women in underprivileged communities.

In fact, there is a plethora of literature documenting the experiences of persons with disabilities; however, there are a dearth of studies on the experiences of black deaf women in accessing healthcare services amongst other things. Chapple (2019) aptly lamented that black deaf women are largely understudied, and their voice is almost invisible in all areas of scholarship. This author premises that when talking about the lived experiences of black deaf women, fundamentally, intersectionality plays a significant role, in that, intersectionality brings to the fore the ways marginalised identities interact to shape multiple dimensions of personhood and social location. For marginalised groups, intersecting identities are realised in terms of race, gender, class, sexuality and disability, and often, these manifest simultaneously (Chapple 2019). In the case of deaf women, three identities are at play-race, gender and disability. Deafness crosses the barriers of gender, ethnicity, age, and economic status (Sporek 2014), thereby placing deaf women, more specifically black deaf women (Chapple 2019) at a risk for marginalisation. Consequently, Chapple (2019) asserted that in disability scholarships, black deaf women are often excluded, and when they are included, only one or two of the three identities are focused on.

Attesting to the exclusion of black deaf women in disability scholarship, in sub-Saharan Africa (SSA), there are a dearth of studies on the lived experiences of such women. A quick literature search on the inclusion of these women (assumed as not categorically expressed) revealed six studies, four of which have been conducted in South Africa. The first study by Ismail and Henderson (2014) investigated the experiences of social exclusion amongst young deaf adults, particularly their beliefs and perceptions of human immunodeficiency virus/acquired immune deficiency syndrome (HIV/AIDS). Primarily, this study focused on young deaf adults; however, there was a deliberate effort to include young deaf women, as out of 92 participants, 48 were female. The second study by Senne (2016) highlighted black deaf women’s lived experiences of their constitutional rights in South Africa. In this study, Senne (2016) discussed intersectionality as denoting the ways in which race and gender interact to shape the multiple dimensions of Black women’s employment experiences. True to the observations by Chapple (2019), Senne (2016) focused on two identities – race and gender. The last four studies by Gichane et al. (2017), Senayah et al. (2019), and Adigun and Mngomezulu (2020) addressed access to healthcare services for women, although Senayah and Kritzinger included males in their studies. Gichane et al. (2017) and Adigun and Mngomezulu (2020) documented pregnancy experiences of deaf women. Specifically, Gichane et al. (2017) explored the utilisation of maternity services and pregnancy outcomes amongst deaf women in Cape Town, South Africa, whilst Adigun and Mngomezulu (2020) explored the experiences and satisfaction of pregnant deaf women with antenatal care in Nigeria. Senayah et al. (2019) focused on accessibility of healthcare services by young deaf adolescents in Ghana. Kritzinger et al. (2014) explored other factors that potentially hamper access to healthcare services for deaf people. Whilst these studies are not concerned with intersectionalities, save for the study Senne (2016), they, however, focused on the fundamental right – access to healthcare for women with disabilities. This is the focus of the current study, access to healthcare services for deaf women in Johannesburg, South Africa.

Even in these developed country contexts, there is still a call to provide better access to primary care for persons with deafness (Emond et al. 2015). Across the world, particularly in the developing country contexts, there is, therefore, a need for identifying the requirements of deaf women and commit to integrate their needs into primary health systems as proposed by Tomlinson et al. (2009). In the light of this need, this study, therefore, aimed at exploring the communication experiences of black deaf South African women accessing healthcare services at public healthcare institutions in Gauteng. As such, this study aims to answer the following question: what are the communication experiences of black deaf and/or HoH when accessing public healthcare services

## Methodology

This study sought to gain insights into the communication experiences of women who are deaf and/or HoH when accessing public healthcare services in hospitals in Johannesburg. In order to achieve this aim, a qualitative phenomenological research design was utilised to capture the lived experiences of a sample of these women. This is a qualitative research study that focuses on the insight, discovery and understanding from the participant’s perspective (Merriam 2002) on a specific issue. A purposive sampling strategy was adopted because it allowed the researchers to collect a sample from a population who met the inclusion criteria of the study and were also accessible to the researcher (Burns & Grove 2009), thereby making it time and cost-effective (Yin 2016) and feasible for both the researcher and participants. For inclusion in this study, participants had to be women who are deaf or HoH, who use public healthcare services and who were between the ages of 22 years and 50 years at the time of the study. This age range was chosen based on the assumption that women of this age are generally mobile, and thus, able to attend public health care facilities, and that they are likely to have a good understanding of their needs and expectations in terms of healthcare (De Haan, Dennil & Vasuthevan 2005). Subsequently, a sample size of 10 participants was recruited (Table 1). Whilst this sample size may be considered small, Saini and Shlonsky (2012) stated that for qualitative interviews, this sample size is sufficient. Additionally, in this study, saturation was obtained by Saunders et al. (2018). Therefore, after the ninth interview, there was no new information from participants.

**TABLE 1:** Participant profiles.

Number of participants (10)	Race	Age (Years)	Level of education	Occupation	Deaf or hard of hearing and mode of communication or language
Participant 1	Black	39	University	Director	Deaf – using SASL
Participant 2	Black	32	University	Clerk	Deaf – using SASL
Participant 3	Mixed race	42	College	South African sign language teacher	Deaf
Participant 4	Black	29	University	Training to be a social auxiliary worker	Deaf – using SASL
Participant 5	Black	22	Grade 12	Training to be a social auxiliary worker	Deaf, wears hearing aids – uses English, Zulu and South African sign language.
Participant 6	Indian	31	Grade 10	Unemployed	Deaf, uses speech reading and speech as well as sign language.
Participant 7	Black	28	Technical skills training in high-school	Factory worker	Deaf – using SASL
Participant 8	Black	32	Grade 10	Unemployed	Deaf – SASL
Participant 9	Black	32	Grade 8	Shop attendant	Deaf – SASL
Participant 10	Black	36	University	Bookkeeper	Deaf – SASL

SASL, South African Sign Language.

Data were collected through one-on-one, semi-structured interviews to gather information from participants who met the inclusion criteria mentioned above, and who have personal experiences, attitudes, perceptions and beliefs related to the topic of interest – access to healthcare services for woman who are deaf and/or HoH (DeJonckheere & Vaughn 2019). The interview questions were developed by the researchers and were deductive in nature, as they were based on available studies on access to healthcare services. Interviews were conducted in English and SASL. The researcher was not fluent in SASL; therefore, services of two professional and registered SASL interpreters were enlisted. The first interpreter was available for one day only and conducted the first three interviews. Consequently, a second interpreter was recruited and conducted the remaining interviews. Taking into account that sign language is a visual language, as recommended by Tye-Murray (2009), the interviews were conducted where there was good lighting and away from background noise. For HoH participants, the researcher spoke naturally and clearly, and avoided covering her mouth whilst speaking. Additionally, the researcher ensured that the interviewer and interpreter were facing the participant at all times during the interview. Being cognisant of the importance of trustworthiness, although interpreting was performed by two different interpreters, these interpreters are professionals, meaning that they are aware of the rules governing their role as language brokers when interpreting. Furthermore, all the interviews were conducted by the same researcher who also followed the same procedural checklist, thereby, minimising the possibility of compromising the integrity of the data collection process as two interpreters were used.

Interviews were conducted at Deaf Federation of South Africa (DEAFSA) Gauteng Regional offices situated in the Johannesburg central business district. Deaf Federation of South Africa is a non-profitable organisation that acts as the national research, information and community action organisation on behalf of the Deaf community in South Africa. The individual interviews were conducted at the Director’s office for confidentiality purposes. Thematic analysis was used to analyse the transcribed data, which involved iterative reading of data to identify patterns and themes emerging from the data (Braun & Clarke 2013). Iterative reading of data provides rich and multifaceted material that increases the rigour of the data (Wells 2007). Data were analysed using the steps recommended by Creswell (2012).

In order to ensure that the research tool generated appropriate findings, a pilot study was conducted, which is an initial, preliminary study conducted to analyse the feasibility of a study in order to improve the research design and refine aspects of the final study (Yin 2016). A pilot study was conducted with the first participant who met the inclusion criteria and consented to participate. This study yielded no modifications to interview questions, thereby deeming the study feasible and did not warranty any refinement. As this study had a small sample size, the pilot study findings were included in the main study. The inclusion of pilot studies in main studies is supported by Kim (2011), who maintains that in studies with small sample sizes, pilot studies may be included to augment the sample size of the main study. To address any bias or subjectivity in collecting, handling and analysing data obtained in this study, reflexivity and bracketing were applied. This was achieved by making use of a peer reviewer who assisted in reflecting on the interpretations and analysis of the data. Peer debriefing or reviewing entails having regular meetings with peers who are not part of the research study in order to identify the researcher’s blind spots, information and results, which may have been missed by the researcher, and aspects of trustworthiness, which may have not been upheld accidentally (Flick 2009). Additionally, member checking was conducted after every interview to confirm that the interviewer captured the essence of what was said during the interview. Table 2 reflects the themes and subthemes that emerged from data collected from interviews with participants.

**TABLE 2:** Themes and subthemes from interviews with participants.

Themes	Subthemes	Excerpts from participants
Communication barriers	Compromised quality of care	‘For HIV [*human immunodeficiency virus*] testing, there should be pre-counselling and then after-counselling they get their results. But because the person is deaf, they can’t advise them, so they just test them and send them home. And what if it’s positive? The Deaf woman may think it’s a good thing, they may not understand they have HIV … most deaf women die from HIV/AIDS [*human immunodeficiency virus/acquired immunodeficiency syndrome*] because they have no knowledge because of the communication barrier’. (Participant 3, teacher, 42 years old)
		‘For example, I try to explain that my stomach is aching, and they don’t understand. There are different pains, it could be a stomach-ache or there may be cramps or it’s just aching as if something is pulling, like a “storm” pain in your stomach, so there are different pains. So, if I just point out that my stomach is sore they don’t really know which type of pain. They just say “oh” and give me medication. So, I don’t feel satisfied. I just leave and take the medication and go home. They can’t really help me … they don’t understand. It’s a problem’. (Participant 7, factory worker, 28 years old)
	Infringement on participants’ right to confidentiality	‘No, I don’t want one, no! The nurses and the doctors have to sign; I don’t want an interpreter’. (Participant 4, social auxiallary worker, 29 years old)
		‘Remember, deaf people also want confidentiality’… the health worker or the doctor or nurse should know sign language because it will be easier to communicate with a deaf person one-on-one, never a third person, because with an interpreter now there’s a third person, you never know, maybe there’s no confidentiality and sometimes deaf women’s rights are violated because the interpreter is always there’. (Participant 2, clerk, 32 years old)
		‘When I go to the doctor, it’s very private there and I prefer to go alone. But because of the communication barrier, there needs to be an interpreter there for communication, so we need more interpreters here in South Africa … But the interpreter must be qualified; they must have a license … not just any interpreter from outside who never learned interpreting. You need a professional interpreter … because some interpreters are not professional, they don’t have confidentiality. They should know the code of ethics … and know all of the rules and then they can’. (Participant 7, factory worker, 28 years old)
	Communication barriers negatively influenced participants’ decisions to access healthcare services	‘I am no longer visiting hospitals, I’m not happy about the service, so I’m fine, I’ll just stay at home’. (Participant 5, training social auxiliary worker, 22 years old)
		‘If there’s no sign language they [*health professionals*] will never understand … I feel very discouraged about that and discouraged to go and seek for healthcare services’. (Participant 2, clerk, 32 years old)
Accommodation that is not accommodative	Lack of sign language skills amongst healthcare professionals.	‘I expect the nurses and doctors to respect deaf people and their culture and have a good attitude towards us … I expect them to learn some basic sign language, even if it’s just “hello, how are you” I will be happy … they can maybe even appoint a deaf person to teach them sign language’. (Participant 6, unemployed, 31 years old)
		‘It’s important that they [*health care professionals*] learn how to communicate with deaf people, the world is not only for hearing people … it’s for all people’. (Participant 9, shop attendent, 32 years old)
	Use of writing to foster communication.	‘Even if I write sometimes there is a communication problem, even if I sign to the doctor … obviously there will be a communication problem also. By writing and signing there is a communication barrier’. (Participant 1, director, 39 years old)
		‘They just want us to write it down. For me this is okay, I can write and read, but other deaf people can’t read or write so how will they communicate and access help?’ (Participant 4, social auxiliary worker, 29 years old)
	The use of lip reading to foster communication	‘[… *T*]hey just talk to me and that is silly, it is a silly thing, they will keep on talking and I can’t hear … I am deaf. They want me to lip read’. (Participant 7, factory worker, 28 years old)
		‘Some of them [*nurses*] force you to talk, they say “look at my lips, you can lip read mos!” so it’s very bad’. (Participant 10, bookkeeper, 36 years old)
	Current accommodations are discriminatory.	‘I deserve fair treatment, the same as hearing people, because now when hearing people communicate, they don’t have to write! So why should I write? And I’m not even good at English, so why should I?.’ (Participant 10, bookkeeper, 36 years old)
		‘Hearing people have little challenges. They don’t experience the same challenges as deaf people. For deaf people there are a lot of challenges including communication, but for hearing people there’s less challenges. Everything runs smoothly. But now when I go there, from the time I arrive and wait in the queue, I already have a problem because they will have a file there and will just call my name so I can’t hear what he/she is saying’. (Participant 8, unemployed, 32 years old)
	Current accommodations negatively impact the confidence levels of deaf women.	‘When other deaf women visit the clinic, their experience can be even worse than mine and this is because I can talk, and I can use lip-reading a bit and I am independent. I can go to the clinic myself. But other deaf women, they cannot stand up for themselves … they can’t write, they can’t read, and it gets very worse, they get oppressed’. (Participant 6, unemployed, 31 years old)
		‘[*G*]oing to the hospital and clinic, waiting in the queues and interacting with health professionals was better for me, because I am very confident and know what I need, but other deaf women … they just sit there the whole day without any help. The people working there don’t assist them’. (Participant 8, unemployed, 32 years old)
Negative attitudes of healthcare professionals	-	‘They [*health professionals*] have a very bad attitude, some of them will never help you, you just wait there for the whole day and you end up going home without any assistance … they become very difficult when they see that you’re a deaf person and they don’t know how to help you’. (Participant 2, clerk, 32 years old)
		‘Some have bad attitudes, but some are very good, they help me and I’m happy. The nurses, they come and make sure that everything is fine, and they explain everything to me very well. But some, they just get there and say, “we finished, please go home.” Some explain nicely, but some are bad … they’re very different people’. (Participant 4, training auxillary social worker, 29 years old)

### Ethical considerations

Ethical clearance to conduct the study was obtained from the Department of Speech Pathology and Audiology Internal Ethics Committee (STA_2016_26). The following ethical considerations were exercised whilst conducting this study: all participants provided informed consent to participate in the study. Confidentiality was ensured as participants’ information was kept anonymous and private. All identifying information of participants was removed from records. Whilst anonymity was not guaranteed because of interviews being conducted at DEAFSA offices, in the final report, participant’s identifying information was removed as participants were assigned numbers to anonymise them. Additionally, participants were not coerced into participating in the research study, and the researcher did not intrude on the participant’s time, space and personal lives (Lichtman 2013). Participants were informed that they could withdraw from the study any time without any penalties or negative consequences.

## Discussion

The findings of this study confirm the alienating, exclusion, marginalisation, discrimination, invisibility, lack of independence and autonomy of women who are deaf, which authors such as Chapple (2019) and Purcell (2014) have mentioned, albeit that these studies were not focused on access to healthcare services.

It is evident from the study as well as from the literature that communication is the most prominent barrier to accessing healthcare services for persons who are deaf (Chaveiro, Porto & Barbosa 2009; Kritzinger et al. 2014; Kuenburg et al. 2016; Orrie & Motsohi 2018). Subsequently, communication barriers influence whether healthcare will be accessed by persons who are deaf (Bentes, Vidal & Maia 2011). Haricharan et al. (2013) further posited that communication barriers impede on the right to access as it is impossible to talk about the right to healthcare for persons who are blind without considering the imperative role of language in achieving this right. The extent of compromised patient care as a result of communication barriers between healthcare professionals and persons who are deaf is also captured in Haricharan et al. (2013), where communication barriers resulted in informational gaps, which, in turn, had dire effects on the diagnosis, treatment and standard of care afforded to a woman who is deaf in Western Cape.

Communication challenges are a result of the gaps in sign language skills amongst healthcare professionals treating women who are deaf. The need for sign language training for healthcare professionals was confirmed in studies conducted by Kritzinger et al. (2014), Masuku (2020), Orrie and Motsohi (2018). Orrie and Motsohi (2018) specifically mentioned that the use of written notes and lip reading is a resourceful means of facilitating communication between healthcare professionals and persons who are deaf. Richardson (2014) confirmed the use of written notes and lip reading as methods mostly used as means of communication between patients who are deaf and healthcare professionals; however, he highlights that these methods are unfortunately not effective. In particular, lip reading often presents with inaccuracies of speech reading (Chaveiro et al. 2009; Richardson 2014), with some persons who are deaf not even being able to lip read (Orrie & Motsohi 2018), whilst written notes were said to be time consuming and depended on the literacy levels of the patient (Chaveiro et al. 2009). Written notes, therefore, negatively influenced the confidence levels of women who are deaf who had low literacy levels (Kritzinger et al. 2014).

The use of an interpreter is a common practice and has been advocated for in cases where there is a language barrier between the patient and the healthcare professional (Weiner & Rivera 2004); for participants in this study, it did take away their autonomy and confidentiality, a finding that was collaborated by Orrie and Motsohi (2018). In order to migrate such challenges, it is advisable that healthcare professionals employ the services of a trained interpreter who is well versed with the culture and context, and who is signed to confidentiality (Weiner & Rivera 2004).

An overwhelming majority of studies have reported on the negative attitudes of healthcare professionals towards persons with disabilities (Badu, Opoku & Appiah 2016; Devkota et al. 2017; Khan et al. 2016; Masuku 2020; Shakespeare & Kleine 2013). Persons who are deaf, therefore, also experience these negative attitudes from healthcare professionals (Orrie & Motsohi 2018). Healthcare professionals are reported to possess preconceived prejudices about persons who are deaf (Orrie & Motsohi 2018). As a result, healthcare professionals subject persons who are deaf to bad experiences in healthcare facilities. One of the bad experiences include the calling of patients over voice, making it difficult for them to know when it is their turn (Ubido et al. 2002). This practice by healthcare professionals is unfortunately interpreted by persons who are deaf as being ignored.

## Conclusion

This research study sought to explore the communication experiences of deaf South African women accessing healthcare services at public healthcare institutions. The evidence gathered from this study confirms that black deaf women experience communication difficulties when accessing healthcare services, a basic human right. These communication challenges unfortunately have clinical and ethical implications on the standard of care that is received by this population when they visit healthcare facilities. They further create a sense of isolation and discrimination by the healthcare community amongst Black women who are deaf and those who are not deaf. These findings have implications for policymakers, as well as the healthcare professionals who interact with these women. Therefore, there is a need for evaluating the efficacy and the effectiveness of the policies and legislation concerned with access to healthcare for persons with disabilities in South Africa. There is, furthermore, a need to advocate for disability training programmes that should be rolled out in healthcare facilities to educate healthcare professionals on specific disabilities and also provide practical strategies to ensure an environment that facilitates reasonable accommodation for persons with disabilities.
